# Biological insights from SMA-extracted proteins

**DOI:** 10.1042/BST20201067

**Published:** 2021-06-10

**Authors:** Lucas Unger, Alejandro Ronco-Campaña, Philip Kitchen, Roslyn M. Bill, Alice J. Rothnie

**Affiliations:** College of Health and Life Sciences, Aston University, Birmingham B4 7ET, U.K.

**Keywords:** cryo-electron microscopy, membrane proteins, protein complexes, protein–lipid interactions, SMALP

## Abstract

In the twelve years since styrene maleic acid (SMA) was first used to extract and purify a membrane protein within a native lipid bilayer, this technological breakthrough has provided insight into the structural and functional details of protein–lipid interactions. Most recently, advances in cryo-EM have demonstrated that SMA-extracted membrane proteins are a rich-source of structural data. For example, it has been possible to resolve the details of annular lipids and protein–protein interactions within complexes, the nature of lipids within central cavities and binding pockets, regions involved in stabilising multimers, details of terminal residues that would otherwise remain unresolved and the identification of physiologically relevant states. Functionally, SMA extraction has allowed the analysis of membrane proteins that are unstable in detergents, the characterization of an ultrafast component in the kinetics of electron transfer that was not possible in detergent-solubilised samples and quantitative, real-time measurement of binding assays with low concentrations of purified protein. While the use of SMA comes with limitations such as its sensitivity to low pH and divalent cations, its major advantage is maintenance of a protein's lipid bilayer. This has enabled researchers to view and assay proteins in an environment close to their native ones, leading to new structural and mechanistic insights.

## Introduction

Membrane proteins carry out a wide range of critical functions and are key targets for drug discovery [1]. However, their location embedded within a lipid bilayer has made them more difficult to study than soluble proteins. To investigate structure or function in detail the protein typically needs to be removed from the complex environment of a biological membrane. Traditionally this has been carried out using detergents, which disrupt the lipids and form micelles around the hydrophobic regions of a membrane protein to maintain them in solution [2]. Dodecylmaltoside (DDM) is one of the most commonly used detergents for membrane proteins and has yielded much success [3]. However, detergents do pose certain challenges. There is a fine balance between effective solubilisation and denaturation/destabilisation of the protein, and finding the optimal detergent can be a lengthy process [4]. The detergent micelle does not fully mimic the environment of a lipid bilayer, meaning lateral pressure and/or key interactions between the protein and specific lipids are lost [5–7]. Therefore, much effort has been made to improve the detergents used to increase protein stability [4]. Alternative systems, such as amphipols or nanodiscs, have been developed which better mimic the natural environment of a protein, but these still require initial detergent solubilisation [8,9]. In 2009 the use of styrene maleic acid co-polymers (SMA) for the effective extraction/solubilisation of membrane proteins was first reported [10]. SMA is an amphipathic polymer comprising hydrophobic styrene moieties and hydrophilic maleic acid groups, typically in a ratio of 2 : 1 or 3 : 1 styrene:maleic acid. SMA inserts into membranes and forms small discs of membrane surrounded by the polymer, termed SMA lipid particles (SMALPs) [11]. They have also been referred to as lipodisqs [12], native nanodiscs [13] and PoLiPa (polymer lipid particles) [14]. Membrane proteins extracted within SMALPs retain their natural lipid bilayer environment, yet are small and soluble and amenable to many different downstream techniques [15]. Encapsulated proteins with an affinity tag can be easily purified using affinity chromatography [16,17]. Once formed, the SMALPs are stable and do not require buffers to be supplemented with free SMA, unlike detergents where the concentration of detergent must be kept above the critical micelle concentration (CMC) for all downstream processes [15]. SMA has been shown to be effective for membrane protein solubilisation from a wide range of different expression systems, including bacteria, insect cells, yeast, mammalian cells and plant cells (Table 1). Like any new technique, there are some limitations to the use of SMA. One of these is a sensitivity to pH below 7 or to divalent cations, which can be problematic for proteins which require more acidic conditions or divalent cation binding for function, such as ATP hydrolysis [18,19]. Another issue is encountered with free excess SMA which can interfere with effective binding to affinity resins or antibodies and other downstream procedures [15,20–22]. As a result of these limitations, numerous polymer modifications are being investigated [23,24], including DIBMA (diisobutylene maleic acid), which has an aliphatic group in place of styrene, and has been shown to generate slightly larger discs, which are more tolerant to divalent cations [25,26]. Despite the limitations, application of SMA polymer to the extraction and purification of membrane proteins has grown substantially over the last 10 years. From initial proof of concept studies, to the application to a wide range of different protein families and cell systems and numerous downstream analysis techniques. In this review, we will examine the biological insights that have been made using SMA (and related polymer)-extracted proteins. This will be divided into three main themes: structural insights, functional insights and protein–lipid interactions.

**Table 1:** Examples of different proteins and expression systems successfully solubilised using polymers

Expression organism	Membrane type	Target protein	Polymer	Reference
*E. coli*	Total membrane	BmrA	SMA2000, SMA3000, SZ25010, SZ30010, DIBMA	[18,19,26]
		ZipA	SMA2000, SMA3000, SZ25010, SZ30010, DIBMA	[16,18,19,26,28]
		KcsA	SMA2000	[13]
		AcrB	SMA2000	[21,29,30]
		bacteriorhodopsin	SZ25010	[31]
		SecYEG	SMA3000	[32]
		KimA	SZ30010	[33]
		YnaI	DIBMA	[34]
		GlpG	DIBMA, SZ25010, SZ30010, SMA2000	[35,36]
*Rhodobacter sphaeroides*	Total membrane	Cytochrome bc1	SZ30010	[37]
*Flavobacterium johnsoniae*	Total membrane	Alternative complex III	SMA3000	[38]
*Thermosynechococcus elongatus*	Thylakoid	Photosystem I	SMA1440	[39]
*Sacchromyces cerevisiae*	Mitochondrial	Cytochrome oxidase	SMA EF30	[40]
	Total membrane	Wsc1	SMA3000	[41]
*Pichia pastoris*	Total membrane	Adenosine 2A receptor	SMA2000	[42]
		Melatonin receptor	SMA2000	[43]
		CD81	SMA2000	[22]
*Sf9* insect cells	Total membrane	hENT1/SLC29A1	SZ30010	[44]
		MRP4/ABCC4	SMA2000	[45]
		α1 glycine receptor	SZ30010	[46]
*High five* insect cells	Total membrane	P-glycoprotein/ABCB1	SMA2000	[47]
HEK293T cells	Total membrane	Dopamine receptor	SMA3000	[48]
		ABCG2	SMA2000	[49]
		Acid-sensing ion channel isoform 1	SZ30010	[50]
Hela cells	Whole cells	Total membrane solubilisaton	SZ30010	[51]
Red blood cells	Red blood cell ghosts	Rh complexes	SMA3000	[52]
Jurkat cells	Total membrane	Numerous cell surface proteins	SMA3000	[53]
Hamster brain	Tissue homogenate	Prion protein	SZ25010, SZ30010	[54]
*Spinacia oleracea*	Chloroplast thylakoid	Total protein	SMA1440, SZ25010, SZ30010, SMA2000	[27]
*Sorghum bicolor*	Total membrane	Dhurrin catalysing metabolon	SMA2000	[55]

SMA2000, SMA3000, SMA EF30 and SMA1440 polymers are from Cray Valley. SZ30010 and SZ25010 are from Polyscope. DIBMA is from BASF. SMA3000, SMA EF30 and SZ25010 have a styrene:maleic acid ratio of ∼3 : 1 whilst SMA2000 and SZ30010 have a ratio of 2 : 1 and 2.3 : 1, respectively. SMA1440 is a partially esterified variant of SMA [27]. DIBMA is a 1 : 1 alternating polymer of diisobutylene and maleic acid.

## Structural insights

**Figure 1.**
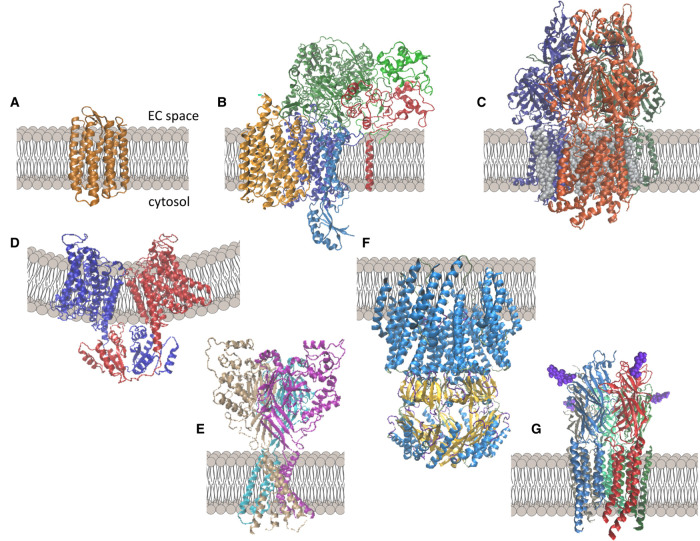
Example structures obtained using polymer extracted proteins. (**A**) Haloquadratum Walsbyi bacteriorhodopsin (PDB ID 5ITC) [31]. (**B**) Alternative complex III (PDB ID 6BTM) [38]. (**C**) AcrB (PDB ID 6BAJ) [21]. (**D**) KimA (PDB ID 6S3K) [33]. (**E**) ASIC1 in the low pH desensitized state (PDB ID 6VTK) [50]. (**F**) YnaI in the closed state (PDB ID 6ZYD) [34]. (**G**) Glycine receptor (GlyR) with bound glycine (purple) in the super-open state (PDB ID 6PM4) [46].

Approaches for the determination of high-resolution protein structure include X-ray crystallography, NMR and cryo-electron microscopy (cryo-EM). Broecker et al. reported the first X-ray structure of an SMA-extracted membrane protein in 2017 using a lipidic cubic phase crystallisation of a bacterial rhodopsin [31] (Figure 1A). Near-atomic resolution (2.0 Å) was observed which was on a comparable level with the detergent-purified receptor in DDM micelles. The concept of being able to go from cell membrane to purified protein and then crystal formation, whilst always having a lipid environment, seemed highly promising, yet to date no further X-ray crystallography structures of SMALP purified proteins have been determined.

NMR based structural studies of membrane proteins can often be challenging simply due to their size, however solid-state NMR of proteins within SMALPs does show some promise for the future. It has been demonstrated that proteins encapsulated in SMALPs magnetically align well and can be used to determine structure [56]. However, it should be noted that this was for relatively small membrane proteins where the structure had previously been determined by NMR, and did not use SMA extraction direct from the expression system. Solid state NMR data has also been obtained for the zinc diffusion facilitator CzcD, but the structure could not be resolved due to challenges in assigning peaks [20].

The most successful approach for determining the structure of membrane proteins within SMALPs has undoubtedly been cryo-EM. The applicability of cryo-EM to SMALP purified proteins was first demonstrated with a low resolution (35 Å) structure of P-glycoprotein/ABCB1 [47] and this was followed by a negative stain EM 23 Å structure of AcrB [30], but these were both ‘proof of concept’ studies of proteins for which the structure had already been determined. The major breakthrough came with the structure of Alternative Complex III (ACIII) from *Flavobacterium johnsoniae* [38] (Figure 1B). This was the first time the structure of this protein, which is a key component of the respiratory and/or photosynthetic electron transport chains of many bacteria [57,58], had been determined. The 3.4 Å resolution cryo-EM map enabled the creation of an atomic model for its six subunits for more than 90% of the sequence. The structure showed, for the first time, the presence of N-terminal triacylated cysteine lipid anchors, as well as the presence of some ordered annular lipids. Both the single enzyme and the functional supercomplex with an *aa*_3_-type cytochrome *c* oxidase was determined, providing details of the protein–protein interactions in this supercomplex. With a size of 464 kDa, the supercomplex is to date the biggest protein extracted in SMALPs.

A 3.2 Å resolution structure of SMALP-encapsulated AcrB was also determined in 2018 by cryo-EM [21] (Figure 1C). Although the structure itself was already known, what this study showed for the first time was the presence of 24 lipids in a central cavity at the trimeric threefold axis, corresponding to a small triangular patch of lipid bilayer. Thus, the SMA approach was able to retain structural elements of the lipid bilayer that are lost when using detergents. This central cavity containing lipids has also been observed subsequently for AcrB reconstituted within a Saposin disc system [59].

A structure of the KUP (K^+^ uptake) family transporter KimA at 3.7 Å resolution was determined within SMALPs [33] (Figure 1D). It was shown to form a homodimer, as expected, but highlighted the regions involved in stabilising this dimeric interaction as well as the likely locations of the potassium binding sites. This contributed to a proposed model for the mechanism of proton-coupled potassium transport by KimA. However, it should be noted that the structure displayed a tilting of the two transmembrane domains towards each other, which caused curvature of the SMALP/surrounding membrane (Figure 1D). Molecular dynamics simulations suggest the protein moves between this tilted state and a more upright state, but the upright state was not observed in the cryo-EM images. The authors postulate this is due to loss of lipid during purification, but it is unclear how or why lipid was lost from the SMALP, or if this is a problem observed elsewhere.

Cryo-EM structures of chicken acid-sensing ion channel (ASIC1) at both pH7 (low pH desensitised) and pH8 (high pH resting state) were determined, at resolutions of 2.8 Å and 3.7 Å respectively [50] (Figure 1E). Although the main structural architecture resembled that of previously determined detergent solubilised structures, the SMA-encapsulated proteins showed strong protein density corresponding to the amino terminal residues for the first time. The pre-transmembrane helix 1 region formed a reentrant loop which may be important for ion permeation. It seems that the retained lipid environment when using SMA was important for preserving the complete protein structure.

The mechanosensitive channel YnaI was isolated from *E.coli* using DIBMA rather than SMA, and it's structure determined by cryo-EM at a resolution of 3.0 Å [34] (Figure 1F). The overall structure was conserved with respect to the related MscS protein, but it was shown to have two additional transmembrane helices per subunit that extend the sensor paddle compared with MscS, although it should be noted that the resolution of these helices was only sufficient for the backbone to be modelled. The structure of YnaI following opening in the presence of LPC was also investigated, and produced three different conformations, although these were all at lower resolution than the closed state structure. Additionally the protein was not obtained directly from *E. coli* but from purified, reconstituted proteoliposomes, which the authors acknowledge contained non-native lipids. The overall shape of the ‘open’ structure is quite different in appearance to the closed state and suggests that the gating mechanism and pore opening of YnaI occur in a completely different way from that seen for MscS. This is possibly related to the presence of lipids in a pocket, for which densities were detected.

Recently the mechanosensitive cell wall sensor Wsc1 was successfully extracted with SMA copolymer from *S. cerevisiae* [41]. The negative stain EM structures obtained were of low resolution but depicted two key states of the protein (extended or folded) and since the cell wall represents the main target in antifungal therapy this study provides the starting point for more extensive studies and functional assessments which will be crucial for future drug discoveries.

Finally, multiple structures of the glycine receptor, a ligand-gated ion channel, have been determined by cryo-EM following SMA extraction [46] (Figure 1G). Interestingly the SMALP encapsulated protein showed three different conformations in the presence of the agonist glycine, which matches with electrophysiology data, but contrasts with the protein in nanodiscs where just a single structure was observed. They were also able to obtain structures in the presence of partial agonists and observed a new, partial agonist-bound closed conformation of the receptor, which is proposed to match the long-lived shut states that are seen in single-channel recordings with partial agonists. These physiologically relevant different states of the protein provide insights to the mechanism of the receptor function.

These studies have shown that the combination of cryo-EM and polymer-extracted proteins enables visualisation of protein–protein complexes and protein–lipid interactions, in a range of different protein conformations, providing previously unknown details.

## Functional insights

Many studies of SMALP-encapsulated proteins have carried out established functional assays to show that the proteins within the SMALPs were indeed functioning, and that this was comparable to either detergent solubilised protein or within native membranes. Techniques included radioligand binding assays [42–44,47,60], spectroscopic binding assays [19,30,32,43,47], characteristic spectra [27,40,61], NMR based assays [10,62], and measurement of channel activity [13,63]. One limitation of SMALP structure, with both sides of the membrane freely accessible is the inability to measure vectorial transport. However, several studies have now demonstrated effective reconstitution of proteins from SMALPs or DIBMALPs into proteoliposomes for measurement of transport [60,64,65].

Purification of the rhomboid protease GlpG using DIBMA demonstrated activity more similar to that observed in membranes than could be measured in detergents [35]. Furthermore, the rhomboid protease from Vibrio cholerae VcROM, which is proteolytically unstable in detergents, remained intact in DIBMA. Similarly, the transport activity of reconstituted human serotonin transporter, hSERT, showed a 5-fold higher level of activity when purified within DIBMALPs than with detergent [60].

An example of novel functional activity, that has been demonstrated through the use of SMALPs, is the binding of the neurotransmitter neurotensin to the dopamine receptor, as measured by microscale thermophoresis [48]. Although prior *in vivo* studies had suggested this interaction may occur, this is the first time a specific interaction could be measured *in vitro*. Secondly, the isolation of cyanobacterial Photosystem I within SMALPs enabled the detection of a new ultrafast component in the kinetics of electron transfer, that has not previously been seen in detergent solubilised samples [39].

Polymer extraction of membrane proteins has also enabled the development of new tools to investigate biological processes and functions. Pellowe et al. [66] have utilised DIBMA extraction of *E. coli* membranes combined with SecM-facilitated nascent chain stalling, to develop a way to isolate ribosome-bound nascent chain complexes for membrane proteins within their native lipid environment, for the first time. This has the potential for future structural and biophysical analysis to gain further insight to the mechanism of co-translational membrane protein folding and membrane insertion. Recently, native-mass spectrometry of SMALP-encapsulated proteins has also been demonstrated, enabling investigation of post-translational modifications, association with specific lipids and protein maturation [67]. Fluorescence correlation spectroscopy of SMALP purified proteins has been utilised to develop a new method for measuring ligand binding to membrane proteins, including both an ABC transporter [49], and a GPCR [68]. This approach enables a quantitative, real-time measurement of binding assays with relatively low concentrations of purified protein. The use of SMA-solubilised bacteria as a tool to isolate and study phage-receptor interactions has been investigated [69]. It was found that specific interactions could be detected between different phage and their target bacteria, which led to DNA ejection by the phage. This has great potential for future work to identify phage receptors.

Finally, the ability to obtain structures of multiple states of some proteins via cryo-EM in polymer lipid particles, as described above, has led to a new understanding of the mechanism of proteins such as YnaI and the glycine receptor [34,46].

## Protein–lipid interactions

One of the advantages of the SMALP approach is the co-extraction of membrane proteins with their surrounding lipid bilayer. It is well established that the function of many membrane proteins is dependent on their lipid environment [5,6,70], and analysis of the lipids co-purified with a protein may be able to yield insight into the native local lipid environment of a protein. Although there are examples of lipids retained within detergent-solubilised structures, it is generally considered that most lipids get displaced by detergents. For example, native mass spectrometry of two different bacteriorhodopsins extracted using SMA showed the presence of native ether-linked lipids. For one of the proteins this was also observed when the detergent octyl glucoside was used, but for the other this lipid adduct was lost with detergent [67]. Thus, it seems more likely that lipid-interactions will be maintained when using SMA than conventional detergents. Several studies have reported analysis of the lipid content of purified protein SMALPs using techniques such as TLC [13,32,37,66,71], HPLC [54] and/or mass spectrometry [22,71,72]. Lipids co-purified from *E.coli* with SMA-extracted KcsA were found to be enriched in both cardiolipin (CL), and phosphatidylglycerol (PG) [13], both of which are negatively charged at physiological pH. Similarly, lipids co-purified with the *E. coli* translocon subunit SecYEG were enriched in CL and PG, yet lower in the zwitterionic phosphatidylethanolamine (PE) [32]. The *Rba. sphaeroides* cytochrome *bc*_1_ (cyt*bc*_1_*)* complex was purified using SMA, and the resulting SMALPs were enriched in CL, and low in sulfoquinovosyl diacylglycerol [37]. In contrast analysis of lipids co-purified from *E. coli* with ZipA suggest it preferentially localised to domains that are relatively enriched in PE and low in CL [72]. Similarly SMALPs of purified aquaporin Z from *E.coli* were low in CL [71]. Lipids co-purified with human CD81 expressed in *Pichia pastoris* were low in PC (phosphatidylcholine) and PE species compared with total and SMA-solubilised membranes, but there were no real differences in CL species. The major lipid that co-purified with prion proteins from infected mice and hamsters was PE, which might relate to a role in prion propagation [54]. PC and cholesterol were also high, but PS (phosphatidyl serine) and PI (phosphatidylinositol) were also detected, which can stimulate prion aggregation.

There have been questions about the reliability of identifying co-purified lipids due to the ability of lipids to diffuse between individual SMALPs [73,74]. However, it should be noted that these studies have utilised lipid-only SMALPs, and it is not yet known how the presence of a protein might affect this lipid exchange. Secondly, it would argue that if specific lipids are found with a given protein, that are distinct from the total solubilised membrane, these must represent only the higher-affinity protein–lipid interactions with slow off-rates. It is not yet clear whether SMA (or other polymers) has bias towards solubilising specific lipids. One study reported no differences in lipid content between SMALPs and the originating *E. coli* membrane [72], whereas subtle differences were observed between total membrane and SMA solubilised samples for *Pichia pastoris* expressing CD81 [22]. Lipidomic analysis of nanodiscs generated using a variety of polymers, including SMA and DIBMA, suggested that glycerolipids are strongly enriched and phospholipids de-enriched in polymer-solubilised samples compared with the originating *E. coli* and Jurkat membranes [75]. In model heterogenous phase-separated bilayers, SMA preferentially solubilised lipids in the fluid phase [76], suggesting that biological membrane microdomains with differing fluidity may be differentially soluble in SMA. It was noted when studying solubilisation of whole Hela cells by SMA that organelle membranes were solubilised more quickly than plasma membranes, which might be explained by plasma membrane domains with different properties [51]. Solubilisation of Jurkat cells with SMA gave both large and small SMALPs, with the lipid composition of the large SMALPs suggested to be more representative of lipid rafts [53]. However, even if the polymers do preferentially solubilise certain lipids, this does not explain differences in lipid content between total SMA solubilised membranes and protein-specific SMALPs purified from this sample.

To date only a few studies have utilised SMALPs to investigate the effects of specific lipids on the function or dynamics of the protein. For SecYEG, which was identified in purified SMALPs to be enriched in CL and PG, reconstitution into proteoliposomes with varying lipid contents demonstrated that the presence of at least one of these anionic lipids was crucial for full protein translocation activity [32]. Reading et al. modulated the cellular lipid environment of GlpG by using slightly different *E.coli* strains and/or changing the temperature. This enabled SMA extraction and purification of GlpG with different co-purifying lipids and they then used hydrogen-deuterium exchange mass spectrometry to monitor difference in protein dynamics [36]. They found that differences in PE and PG content had no major impact on GlpG dynamics, however differences in chain length and unsaturation leading to more fluid bilayers did impact GlpG dynamics. SMALPs formed of defined lipid compositions were used to investigate the interactions between lipids and α-synuclein, the main protein involved in Parkinson's disease [77]. The presence of cholesterol was found to both inhibit interaction of α-synuclein with lipids and promote α-synuclein aggregation. Using NMR they were able to start to pinpoint the specific effects of different lipids on specific regions of the protein, to better understand this complex interaction, and found that the parts of the protein affected by cholesterol depended on which other lipids were also present. Finally, the EphA2 receptor tyrosine kinase can dimerise in two different binding modes with different transmembrane helix crossing angles, with distinct impacts on signalling. EphA2 transmembrane peptide was reconstituted into proteoliposomes with different bilayer thickness, to bias the dimerisation towards one of the two binding modes, and these proteoliposomes were solubilised with SMA for single molecule fluorescence studies. Addition of PIP_2_ was found to increase the dimer:monomer ratio only in the thicker bilayer, suggesting that PIP signalling may modulate the pro-oncogenic ligand-independent signalling of EphA2 [78].

As described already, several of the cryo-EM structures of SMALP-encapsulated proteins contained densities corresponding to lipids. For the *E. coli* mechanosensitive channel YnaI solubilised with DIBMA, one lipid molecule was well-resolved, with the phosphate headgroup forming a salt bridge with a lysine residue (K108). Mutation of this lysine (K108L) significantly reduced the pressure required to open YnaI, suggested that lipid coordination at this site is functionally important for pressure sensing [34]. Also, in the cryo-EM structure of the trimeric *E. coli* multi-drug efflux transporter AcrB, the patch of lipids in the central cavity has been suggested to play a role in harmonising the conformational changes of the protein [21]. Previous structural studies of detergent-solubilised AcrB mutants suggested that large inward movements of transmembrane domains into the central cavity were associated with disruption of function. However, in the cryo-EM structure of one of these same mutants solubilised with SMA, the presence of the central bilayer patch was still present and restricted this inward motion. This suggests that these motions were exaggerated by the removal of the cavity lipids in structures of detergent-solubilised AcrB, and that the structural difference between wild-type and mutants of AcrB may be much more subtle [21].

## Conclusions

**Figure 2.**
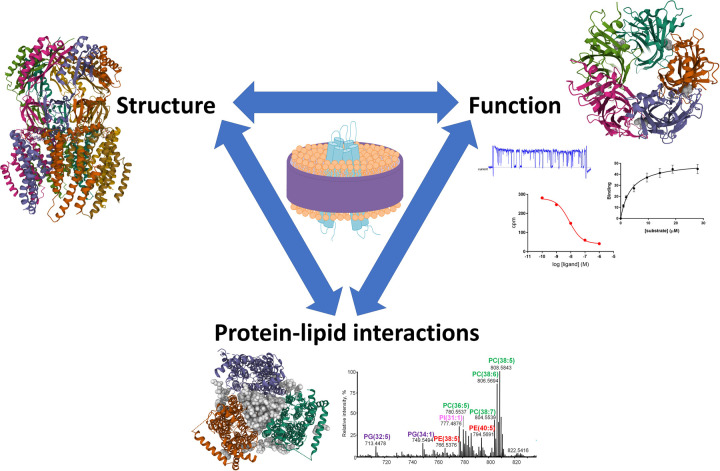
Polymer extraction of membrane proteins retains interactions between proteins and lipids which is important for both protein structure and function. Side view structure of YnaI (PDB ID 6ZYD) [34].Top view structure of GlyR showing bound partial agonist taurine (space filling grey) (PDB ID 6PM0) [46], alongside representative images of various types of functional assays. Bottom view of AcrB trimer showing central lipid filled cavity (space filling grey) (PDB ID 6BAJ) [21], alongside a representative mass spectrum for lipids co-purified with a protein from yeast. Structural images made using Mol*[79].

The SMA approach is not without limitations, the sensitivity to low pH or divalent cations, the problems of free SMA interfering with binding or inhibiting activity, and it remains unclear whether the SMALP structure allows full dynamic function of all proteins or if it might be restrictive in some cases. Therefore, significant effort is being made to improve upon this, with many different polymers being made and tested. However, the maintenance of a lipid environment that SMA extraction of membrane proteins facilitates is arguably the biggest benefit of this approach, and this impacts on both structure and function of proteins (Figure 2). It has enabled researchers to view and assay proteins in a much closer to native format, which has led to new mechanistic insights. The investigation of the specific interactions between lipids and proteins is likely to be an area of interest for some time to come.

## Perspectives

- Extraction/solubilisation and purification of membrane proteins using SMA (and related polymers) overcomes several of the limitations associated with detergents, allows the lipid bilayer environment of the protein to be retained, and is amenable to many downstream techniques.
- High resolution structural determination by cryo-EM of polymer encapsulated proteins has been successful, enabling new knowledge on protein complex formation and the association with lipids.
- SMALPs allow study of the important interactions between membrane proteins and their lipid environment and this will likely be exploited further in the future.

## Open Access

Open access for this article was enabled by the participation of Aston University in an all-inclusive *Read & Publish* pilot with Portland Press and the Biochemical Society under a transformative agreement with JISC.

## Abbreviations

ABC: ATP Binding Cassette
CL: cardiolipin
CMC: critical micelle concentration
DDM: dodecylmaltoside
DIBMA: diisobutylene maleic acid
DIBMALP: DIBMA lipid particle
EM: electron microscopy
GPCR: G protein-coupled receptor
KUP: K^+^ uptake transporters
PC: phosphatidylcholine
PE: phosphatidylethanolamine
PG: phosphatidylglycerol
PI: phosphatidylinositol
PoLiPa: polymer lipid particles
PS: phospahtidylserine
SMA: styrene maleic acid
SMALP: SMA lipid particle

## References

[1] Overington, J.P., Al-Lazikani, B. and Hopkins, A.L. (2006) How many drug targets are there? Nat. Rev. Drug Discov. 5, 993–996 10.1038/nrd2199 17139284

[2] Seddon, A.M., Curnow, P. and Booth, P.J. (2004) Membrane proteins, lipids and detergents: not just a soap opera. Biochim. Biophys. Acta Biomembr. 1666, 105–117 10.1016/j.bbamem.2004.04.011 15519311

[3] Choy, B.C., Cater, R.J., Mancia, F. and Pryor, Jr, E.E. (2021) A 10-year meta-analysis of membrane protein structural biology: detergents, membrane mimetics, and structure determination techniques. Biochim. Biophys. Acta Biomembr. 1863, 183533 10.1016/j.bbamem.2020.183533 33340490 PMC7856071

[4] Hardy, D., Desuzinges Mandon, E., Rothnie, A.J. and Jawhari, A. (2018) The yin and yang of solubilization and stabilization for wild-type and full-length membrane protein. Methods 147, 118–125 10.1016/j.ymeth.2018.02.017 29477816

[5] Dawaliby, R., Trubbia, C., Delporte, C., Masureel, M., Van Antwerpen, P., Kobilka, B.K. et al. (2016) Allosteric regulation of G protein-coupled receptor activity by phospholipids. Nat. Chem. Biol. 12, 35–39 10.1038/nchembio.1960 26571351 PMC4718399

[6] Zoghbi, M.E., Cooper, R.S. and Altenberg, G.A. (2016) The lipid bilayer modulates the structure and function of an ATP-binding cassette exporter. J. Biol. Chem. 291, 4453–4461 10.1074/jbc.M115.698498 26725230 PMC4813473

[7] van den Brink-van der Laan, E., Chupin, V., Killian, J.A. and de Kruijff, B. (2004) Stability of KcsA tetramer depends on membrane lateral pressure. Biochemistry 43, 4240–4250 10.1021/bi036129d 15065868

[8] Zoonens, M. and Popot, J.L. (2014) Amphipols for each season. J. Membr. Biol. 247, 759–796 10.1007/s00232-014-9666-8 24969706 PMC4282167

[9] Denisov, I.G. and Sligar, S.G. (2016) Nanodiscs for structural and functional studies of membrane proteins. Nat. Struct. Mol. Biol. 23, 481–486 10.1038/nsmb.3195 27273631 PMC8934039

[10] Knowles, T.J., Finka, R., Smith, C., Lin, Y.P., Dafforn, T. and Overduin, M. (2009) Membrane proteins solubilized intact in lipid containing nanoparticles bounded by styrene maleic acid copolymer. J. Am. Chem. Soc. 131, 7484–7485 10.1021/ja810046q 19449872

[11] Jamshad, M., Grimard, V., Idini, I., Knowles, T.J., Dowle, M.R., Schofield, N. et al. (2015) Structural analysis of a nanoparticle containing a lipid bilayer used for detergent-free extraction of membrane proteins. Nano Res. 8, 774–789 10.1007/s12274-014-0560-6 31031888 PMC6485620

[12] Orwick-Rydmark, M., Lovett, J.E., Graziadei, A., Lindholm, L., Hicks, M.R. and Watts, A. (2012) Detergent-free incorporation of a seven-transmembrane receptor protein into nanosized bilayer lipodisq particles for functional and biophysical studies. Nano Lett. 12, 4687–4692 10.1021/nl3020395 22827450

[13] Dorr, J.M., Koorengevel, M.C., Schafer, M., Prokofyev, A.V., Scheidelaar, S., van der Cruijsen, E.A. et al. (2014) Detergent-free isolation, characterization, and functional reconstitution of a tetrameric K+ channel: the power of native nanodiscs. Proc. Natl Acad. Sci. U.S.A. 111, 18607–18612 10.1073/pnas.1416205112 25512535 PMC4284610

[14] Hothersall, J.D., Jones, A.Y., Dafforn, T.R., Perrior, T. and Chapman, K.L. (2020) Releasing the technical ‘shackles’ on GPCR drug discovery: opportunities enabled by detergent-free polymer lipid particle (PoLiPa) purification. Drug Discov. Today 25, 1944–1956 10.1016/j.drudis.2020.08.006 32835806

[15] Pollock, N.L., Lee, S.C., Patel, J.H., Gulamhussein, A.A. and Rothnie, A.J. (2018) Structure and function of membrane proteins encapsulated in a polymer-bound lipid bilayer. Biochim. Biophys Acta 1860, 809–817 10.1016/j.bbamem.2017.08.012 28865797

[16] Lee, S.C., Knowles, T.J., Postis, V.L., Jamshad, M., Parslow, R.A., Lin, Y.P. et al. (2016) A method for detergent-free isolation of membrane proteins in their local lipid environment. Nat. Protoc. 11, 1149–1162 10.1038/nprot.2016.070 27254461

[17] Rothnie, A.J. (2016) Detergent-Free membrane protein purification. Methods Mol. Biol. 1432, 261–267 10.1007/978-1-4939-3637-3_16 27485341

[18] Gulamhussein, A.A., Meah, D., Soja, D.D., Fenner, S., Saidani, Z., Akram, A. et al. (2019) Examining the stability of membrane proteins within SMALPs. Eur. Polym. J. 112, 120–125 10.1016/j.eurpolymj.2018.12.008

[19] Morrison, K.A., Akram, A., Mathews, A., Khan, Z.A., Patel, J.H., Zhou, C. et al. (2016) Membrane protein extraction and purification using styrene-maleic acid (SMA) copolymer: effect of variations in polymer structure. Biochem. J. 473, 4349–4360 10.1042/BCJ20160723 27694389

[20] Bersch, B., Dorr, J.M., Hessel, A., Killian, J.A. and Schanda, P. (2017) Proton-detected solid-state NMR spectroscopy of a zinc diffusion facilitator protein in native nanodiscs. Angew. Chem. Int. Ed. Engl. 56, 2508–2512 10.1002/anie.201610441 28128538 PMC5321536

[21] Qiu, W., Fu, Z., Xu, G.G., Grassucci, R.A., Zhang, Y., Frank, J. et al. (2018) Structure and activity of lipid bilayer within a membrane-protein transporter. Proc. Natl Acad. Sci. U.S.A. 115, 12985–12990 10.1073/pnas.1812526115 30509977 PMC6304963

[22] Ayub, H., Clare, M., Milic, I., Chmel, N.P., Boning, H., Devitt, A. et al. (2020) CD81 extracted in SMALP nanodiscs comprises two distinct protein populations within a lipid environment enriched with negatively charged headgroups. Biochim. Biophys. Acta Biomembr. 1862, 183419 10.1016/j.bbamem.2020.183419 32735789 PMC7456796

[23] Hall, S.C.L., Tognoloni, C., Price, G.J., Klumperman, B., Edler, K.J., Dafforn, T.R. et al. (2018) Influence of poly(styrene- co-maleic acid) copolymer structure on the properties and self-assembly of SMALP nanodiscs. Biomacromolecules 19, 761–772 10.1021/acs.biomac.7b01539 29272585

[24] Stroud, Z., Hall, S.C.L. and Dafforn, T.R. (2018) Purification of membrane proteins free from conventional detergents: SMA, new polymers, new opportunities and new insights. Methods 147, 106–117 10.1016/j.ymeth.2018.03.011 29608964

[25] Oluwole, A.O., Danielczak, B., Meister, A., Babalola, J.O., Vargas, C. and Keller, S. (2017) Solubilization of membrane proteins into functional lipid-bilayer nanodiscs using a diisobutylene/maleic acid copolymer. Angew. Chem. Int. Ed. Engl. 56, 1919–1924 10.1002/anie.201610778 28079955 PMC5299484

[26] Gulamhussein, A.A., Uddin, R., Tighe, B.J., Poyner, D.R. and Rothnie, A.J. (2020) A comparison of SMA (styrene maleic acid) and DIBMA (di-isobutylene maleic acid) for membrane protein purification. Biochim. Biophys. Acta Biomembr. 1862, 183281 10.1016/j.bbamem.2020.183281 32209303

[27] Korotych, O., Mondal, J., Gattás-Asfura, K.M., Hendricks, J. and Bruce, B.D. (2019) Evaluation of commercially available styrene-co-maleic acid polymers for the extraction of membrane proteins from spinach chloroplast thylakoids. Eur. Polym. J. 114, 485–500 10.1016/j.eurpolymj.2018.10.035

[28] Lee, S.C., Collins, R., Lin, Y.P., Jamshad, M., Broughton, C., Harris, S.A. et al. (2019) Nano-encapsulated *Escherichia coli* divisome anchor ZipA, and in complex with FtsZ. Sci. Rep. 9, 18712 10.1038/s41598-019-54999-x 31822696 PMC6904479

[29] Parmar, M., Rawson, S., Scarff, C.A., Goldman, A., Dafforn, T.R., Muench, S.P. et al. (2018) Using a SMALP platform to determine a sub-nm single particle cryo-EM membrane protein structure. Biochim. Biophys. Acta 1860, 378–383 10.1016/j.bbamem.2017.10.005 PMC5780298 28993151

[30] Postis, V., Rawson, S., Mitchell, J.K., Lee, S.C., Parslow, R.A., Dafforn, T.R. et al. (2015) The use of SMALPs as a novel membrane protein scaffold for structure study by negative stain electron microscopy. Biochim. Biophys. Acta 1848, 496–501 10.1016/j.bbamem.2014.10.018 25450810 PMC4331651

[31] Broecker, J., Eger, B.T. and Ernst, O.P. (2017) Crystallogenesis of membrane proteins mediated by polymer-bounded lipid nanodiscs. Structure 25, 384–392 10.1016/j.str.2016.12.004 28089451

[32] Prabudiansyah, I., Kusters, I., Caforio, A. and Driessen, A.J. (2015) Characterization of the annular lipid shell of the Sec translocon. Biochim. Biophys. Acta 1848, 2050–2056 10.1016/j.bbamem.2015.06.024 26129641

[33] Tascon, I., Sousa, J.S., Corey, R.A., Mills, D.J., Griwatz, D., Aumuller, N. et al. (2020) Structural basis of proton-coupled potassium transport in the KUP family. Nat. Commun. 11, 626 10.1038/s41467-020-14441-7 32005818 PMC6994465

[34] Flegler, V.J., Rasmussen, A., Rao, S., Wu, N., Zenobi, R., Sansom, M.S.P. et al. (2020) The MscS-like channel YnaI has a gating mechanism based on flexible pore helices. Proc. Natl Acad. Sci. U.S.A. 117, 28754–28762 10.1073/pnas.2005641117 33148804 PMC7682570

[35] Barniol-Xicota, M. and Verhelst, S.H.L. (2018) Stable and functional rhomboid proteases in lipid nanodiscs by using diisobutylene/maleic acid copolymers. J. Am. Chem. Soc. 140, 14557–14561 10.1021/jacs.8b08441 30347979

[36] Reading, E., Hall, Z., Martens, C., Haghighi, T., Findlay, H., Ahdash, Z. et al. (2017) Interrogating membrane protein conformational dynamics within native lipid compositions. Angew. Chem. Int. Ed. Engl. 56, 15654–7 10.1002/anie.201709657 29049865

[37] Swainsbury, D.J.K., Proctor, M.S., Hitchcock, A., Cartron, M.L., Qian, P., Martin, E.C. et al. (2018) Probing the local lipid environment of the *Rhodobacter sphaeroides* cytochrome bc1 and Synechocystis sp. PCC 6803 cytochrome b6f complexes with styrene maleic acid. Biochim. Biophys. Acta Bioenerg. 1859, 215–225 10.1016/j.bbabio.2017.12.005 29291373 PMC5805856

[38] Sun, C., Benlekbir, S., Venkatakrishnan, P., Wang, Y., Hong, S., Hosler, J. et al. (2018) Structure of the alternative complex III in a supercomplex with cytochrome oxidase. Nature 557, 123–126 10.1038/s41586-018-0061-y 29695868 PMC6004266

[39] Cherepanov, D.A., Brady, N.G., Shelaev, I.V., Nguyen, J., Gostev, F.E., Mamedov, M.D. et al. (2020) PSI-SMALP, a detergent-free cyanobacterial photosystem I, reveals faster femtosecond photochemistry. Biophys. J. 118, 337–351 10.1016/j.bpj.2019.11.3391 31882247 PMC6976803

[40] Smirnova, I.A., Sjostrand, D., Li, F., Bjorck, M., Schafer, J., Ostbye, H. et al. (2016) Isolation of yeast complex IV in native lipid nanodiscs. Biochim. Biophys. Acta 1858, 2984–2992 10.1016/j.bbamem.2016.09.004 27620332 PMC9472556

[41] Voskoboynikova, N., Karlova, M., Kurre, R., Mulkidjanian, A.Y., Shaitan, K.V., Sokolova, O.S. et al. (2021) A three-dimensional model of the yeast transmembrane sensor Wsc1 obtained by SMA-Based detergent-free purification and transmission electron microscopy. J. Fungi (Basel) 7, 118 10.3390/jof7020118 33562593 PMC7915640

[42] Jamshad, M., Charlton, J., Lin, Y.P., Routledge, S.J., Bawa, Z., Knowles, T.J. et al. (2015) G-protein coupled receptor solubilization and purification for biophysical analysis and functional studies, in the total absence of detergent. Biosci. Rep. 35, e00188 10.1042/BSR20140171 25720391 PMC4400634

[43] Logez, C., Damian, M., Legros, C., Dupre, C., Guery, M., Mary, S. et al. (2016) Detergent-free isolation of functional G protein-coupled receptors into nanometric lipid particles. Biochemistry 55, 38–48 10.1021/acs.biochem.5b01040 26701065

[44] Rehan, S. and Jaakola, V.P. (2015) Expression, purification and functional characterization of human equilibrative nucleoside transporter subtype-1 (hENT1) protein from Sf9 insect cells. Protein Expr. Purif. 114, 99–107 10.1016/j.pep.2015.07.003 26162242

[45] Hardy, D., Bill, R.M., Rothnie, A.J. and Jawhari, A. (2019) Stabilization of human multidrug resistance protein 4 (MRP4/ABCC4) using novel solubilization agents. SLAS Discov. 24, 1009–1017 10.1177/2472555219867074 31381456 PMC6873219

[46] Yu, J., Zhu, H., Lape, R., Greiner, T., Du, J., Lu, W. et al. (2021) Mechanism of gating and partial agonist action in the glycine receptor. Cell 184, 957–68.e21 10.1016/j.cell.2021.01.026 33567265 PMC8115384

[47] Gulati, S., Jamshad, M., Knowles, T.J., Morrison, K.A., Downing, R., Cant, N. et al. (2014) Detergent-free purification of ABC (ATP-binding-cassette) transporters. Biochem. J. 461, 269–278 10.1042/BJ20131477 24758594

[48] Bada Juarez, J.F., Munoz-Garcia, J.C., Inacio Dos Reis, R., Henry, A., McMillan, D., Kriek, M. et al. (2020) Detergent-free extraction of a functional low-expressing GPCR from a human cell line. Biochim Biophys Acta Biomembr. 1862, 183152 10.1016/j.bbamem.2019.183152 31843475

[49] Horsey, A.J., Briggs, D.A., Holliday, N.D., Briddon, S.J. and Kerr, I.D. (2020) Application of fluorescence correlation spectroscopy to study substrate binding in styrene maleic acid lipid copolymer encapsulated ABCG2. Biochim. Biophys. Acta Biomembr. 1862, 183218 10.1016/j.bbamem.2020.183218 32057756 PMC7156912

[50] Yoder, N. and Gouaux, E. (2020) The His-Gly motif of acid-sensing ion channels resides in a reentrant ‘loop’ implicated in gating and ion selectivity. eLife 9, e56527 10.7554/eLife.56527 32496192 PMC7308080

[51] Dorr, J.M., van Coevorden-Hameete, M.H., Hoogenraad, C.C. and Killian, J.A. (2017) Solubilization of human cells by the styrene-maleic acid copolymer: insights from fluorescence microscopy. Biochim. Biophys. Acta Biomembr. 1859, 2155–2160 10.1016/j.bbamem.2017.08.010 28847501

[52] Desrames, A., Genetet, S., Delcourt, M.P., Goossens, D. and Mouro-Chanteloup, I. (2020) Detergent-free isolation of native red blood cell membrane complexes. Biochim. Biophys. Acta Biomembr. 1862, 183126 10.1016/j.bbamem.2019.183126 31738902

[53] Angelisova, P., Ballek, O., Sykora, J., Benada, O., Cajka, T., Pokorna, J. et al. (2019) The use of styrene-maleic acid copolymer (SMA) for studies on T cell membrane rafts. Biochim. Biophys. Acta Biomembr. 1861, 130–141 10.1016/j.bbamem.2018.08.006 30463696

[54] Esmaili, M., Tancowny, B.P., Wang, X., Moses, A., Cortez, L.M., Sim, V.L. et al. (2020) Native nanodiscs formed by styrene maleic acid copolymer derivatives help recover infectious prion multimers bound to brain-derived lipids. J. Biol. Chem. 295, 8460–8469 10.1074/jbc.RA119.012348 32358064 PMC7307199

[55] Laursen, T., Borch, J., Knudsen, C., Bavishi, K., Torta, F., Martens, H.J. et al. (2016) Characterization of a dynamic metabolon producing the defense compound dhurrin in sorghum. Science 354, 890–893 10.1126/science.aag2347 27856908

[56] Park, S.H., Wu, J., Yao, Y., Singh, C., Tian, Y., Marassi, F.M. et al. (2020) Membrane proteins in magnetically aligned phospholipid polymer discs for solid-state NMR spectroscopy. Biochim. Biophys. Acta Biomembr. 1862, 183333 10.1016/j.bbamem.2020.183333 32371072 PMC8370186

[57] Yanyushin, M.F., del Rosario, M.C., Brune, D.C. and Blankenship, R.E. (2005) New class of bacterial membrane oxidoreductases. Biochemistry 44, 10037–10045 10.1021/bi047267l 16042380

[58] Refojo, P.N., Sousa, F.L., Teixeira, M. and Pereira, M.M. (2010) The alternative complex III: a different architecture using known building modules. Biochim. Biophys. Acta Bioenergetics 1797, 1869–1876 10.1016/j.bbabio.2010.04.012 20416271

[59] Du, D., Neuberger, A., Orr, M.W., Newman, C.E., Hsu, P.C., Samsudin, F. et al. (2020) Interactions of a bacterial RND transporter with a transmembrane small protein in a lipid environment. Structure 28, 625–34.e6 10.1016/j.str.2020.03.013 32348749 PMC7267776

[60] Dilworth, M.V., Findlay, H.E. and Booth, P.J. (2021) Detergent-free purification and reconstitution of functional human serotonin transporter (SERT) using diisobutylene maleic acid (DIBMA) copolymer. Biochim. Biophys. Acta Biomembr. 1863, 183602 10.1016/j.bbamem.2021.183602 33744253 PMC8111416

[61] Swainsbury, D.J., Scheidelaar, S., van Grondelle, R., Killian, J.A. and Jones, M.R. (2014) Bacterial reaction centers purified with styrene maleic acid copolymer retain native membrane functional properties and display enhanced stability. Angew. Chem. Int. Ed. Engl. 53, 11803–7 10.1002/anie.201406412 25212490 PMC4271668

[62] Li, D., Li, J., Zhuang, Y., Zhang, L., Xiong, Y., Shi, P. et al. (2015) Nano-size uni-lamellar lipodisq improved in situ auto-phosphorylation analysis of E. coli tyrosine kinase using (19)F nuclear magnetic resonance. Protein Cell 6, 229–233 10.1007/s13238-014-0129-x 25564343 PMC4348245

[63] Krajewska, M. and Koprowski, P. (2021) Solubilization, purification, and functional reconstitution of human ROMK potassium channel in copolymer styrene-maleic acid (SMA) nanodiscs. Biochim. Biophys. Acta Biomembr. 1863, 183555 10.1016/j.bbamem.2021.183555 33444624

[64] Smirnova, I.A., Adelroth, P. and Brzezinski, P. (2018) Extraction and liposome reconstitution of membrane proteins with their native lipids without the use of detergents. Sci. Rep. 8, 14950 10.1038/s41598-018-33208-1 30297885 PMC6175888

[65] Dutta, D., Esmaili, M., Overduin, M. and Fliegel, L. (2020) Expression and detergent free purification and reconstitution of the plant plasma membrane Na(+)/H(+) antiporter SOS1 overexpressed in pichia pastoris. Biochim. Biophys. Acta Biomembr. 1862, 183111 10.1016/j.bbamem.2019.183111 31678368

[66] Pellowe, G.A., Findlay, H.E., Lee, K., Gemeinhardt, T.M., Blackholly, L.R., Reading, E. et al. (2020) Capturing membrane protein ribosome nascent chain complexes in a native-like environment for co-translational studies. Biochemistry 59, 2764–2775 10.1021/acs.biochem.0c00423 32627541 PMC7551657

[67] Hoi, K.K., Bada Juarez, J.F., Judge, P.J., Yen, H.Y., Wu, D., Vinals, J. et al. (2021) Detergent-free lipodisq nanoparticles facilitate high-resolution mass spectrometry of folded integral membrane proteins. Nano Lett. 21, 2824–2831 10.1021/acs.nanolett.0c04911 33787280 PMC8050825

[68] Grime, R.L., Goulding, J., Uddin, R., Stoddart, L.A., Hill, S.J., Poyner, D.R. et al. (2020) Single molecule binding of a ligand to a G-protein-coupled receptor in real time using fluorescence correlation spectroscopy, rendered possible by nano-encapsulation in styrene maleic acid lipid particles. Nanoscale 12, 11518–11525 10.1039/D0NR01060J 32428052

[69] de Jonge, P.A., Smit Sibinga, D.J.C., Boright, O.A., Costa, A.R., Nobrega, F.L., Brouns, S.J.J. et al. (2020) Development of styrene maleic acid lipid particles as a tool for studies of phage-host interactions. J. Virol. 94, e01559-20 10.1128/JVI.01559-20 32938760 PMC7654272

[70] Rothnie, A., Theron, D., Soceneantu, L., Martin, C., Traikia, M., Berridge, G. et al. (2001) The importance of cholesterol in maintenance of P-glycoprotein activity and its membrane perturbing influence. Eur. Biophys. J. 30, 430–442 10.1007/s002490100156 11718296

[71] Schmidt, V., Sidore, M., Bechara, C., Duneau, J.P. and Sturgis, J.N. (2019) The lipid environment of *Escherichia coli* aquaporin Z. Biochim. Biophys. Acta Biomembr. 1861, 431–440 10.1016/j.bbamem.2018.10.017 30414848

[72] Teo, A.C.K., Lee, S.C., Pollock, N.L., Stroud, Z., Hall, S., Thakker, A. et al. (2019) Analysis of SMALP co-extracted phospholipids shows distinct membrane environments for three classes of bacterial membrane protein. Sci. Rep. 9, 1813 10.1038/s41598-018-37962-0 30755655 PMC6372662

[73] Cuevas Arenas, R., Danielczak, B., Martel, A., Porcar, L., Breyton, C., Ebel, C. et al. (2017) Fast collisional lipid transfer among polymer-bounded nanodiscs. Sci. Rep. 7, 45875 10.1038/srep45875 28378790 PMC5381093

[74] Hazell, G., Arnold, T., Barker, R.D., Clifton, L.A., Steinke, N.J., Tognoloni, C. et al. (2016) Evidence of lipid exchange in styrene maleic acid lipid particle (SMALP) nanodisc systems. Langmuir 32, 11845–11853 10.1021/acs.langmuir.6b02927 27739678

[75] Barniol-Xicota, M. and Verhelst, S.H.L. (2021) Lipidomic and in-gel analysis of maleic acid co-polymer nanodiscs reveals differences in composition of solubilized membranes. Commun. Biol. 4, 218 10.1038/s42003-021-01711-3 33594255 PMC7886889

[76] Dominguez Pardo, J.J., Dorr, J.M., Iyer, A., Cox, R.C., Scheidelaar, S., Koorengevel, M.C. et al. (2017) Solubilization of lipids and lipid phases by the styrene-maleic acid copolymer. Eur. Biophys. J. 46, 91–101 10.1007/s00249-016-1181-7 27815573 PMC5209432

[77] Jakubec, M., Barias, E., Furse, S., Govasli, M.L., George, V., Turcu, D. et al. (2021) Cholesterol-containing lipid nanodiscs promote an alpha-synuclein binding mode that accelerates oligomerization. FEBS J. 288, 1887–1905 10.1111/febs.15551 32892498

[78] Stefanski, K.M., Russell, C.M., Westerfield, J.M., Lamichhane, R. and Barrera, F.N. (2020) PIP2 promotes conformation-specific dimerization of the EphA2 membrane region. J Biol Chem. 296, 100149 10.1074/jbc.RA120.016423 33277361 PMC7900517

[79] Sehnal, D., Rose, A.S., Kovca, J., Burley, S.K. and Velankar, S. (2018) Mol*: Towards a common library and tools for web molecular graphics. MolVA ‘18: Proceedings of the Workshop on Molecular Graphics and Visual Analysis of Molecular Data. 29–33

